# Smog and risk of maternal and fetal birth outcomes: A retrospective study in Baoding, China

**DOI:** 10.1515/med-2022-0489

**Published:** 2022-05-31

**Authors:** Yijing Zhai, Bei Wang, Liqiang Qin, Bin Luo, Ying Xie, Huanyu Hu, Hongzhen Du, Zengning Li

**Affiliations:** Department of Nutrition, Hebei Key Laboratory of Nutrition and Health, The First Hospital of Hebei Medical University, Shijiazhuang, 050031, China; Department of Nutrition and Food Hygiene, School of Public Health, Soochow University, Suzhou, 215000, China

**Keywords:** smog, low birth weight, pregnancy-induced hypertension, gestational diabetes mellitus, premature rupture of membranes

## Abstract

Pregnant women are more susceptible to smog pollution than the general population. This study focused on the association between smog and birth outcomes, considering both pregnant mothers and their offspring. In this retrospective study, conducted in Baoding between 2013 and 2016, we enrolled 842 participants. Birth outcomes were low birth weight (LBW), pregnancy-induced hypertension (PIH), gestational diabetes mellitus (GDM), and premature rupture of membranes (PROM). The overall prevalence of LBW, PIH, GDM, and PROM was 8.2%, 14.8%, 16.5%, and 12.1%, respectively. Compared with lower pollution level, higher pollution level of fine particulate matter (particulate matter with aerodynamics diameter <2.5 μm) (PM2.5), inhalable particle (particulate matter with aerodynamics diameter <10 μm) (PM10), and CO increased the risk of term with LBW. PM2.5, PM10, and NO_2_ increased the risk of PIH during different trimesters, while PM10 increased the risk of PROM during trimester 3. In conclusion, smog significantly affects the risk of adverse birth outcomes by different exposure time windows.

## 1 Introduction

According to the World Health Organization (WHO) air pollution database, China has higher levels of air pollution than Western countries [1]. Less than 1% of China’s 500 largest cities meet the air quality standards. With fast economic growth over the past four decades, the air quality in China, particularly in North China, has relatively deteriorated.

Smog seriously threatens human health and has become a hot topic for research and the public. Pregnant women and fetus are more susceptible to environmental factors, including smog pollution, than the general population. Exposure to PM2.5 (particulate matter with aerodynamics diameter <2.5 μm) in trimester 2 of pregnancy was associated with an increased risk of gestational diabetes mellitus (GDM) [2]. Prenatal exposure of the major air pollutants during the entire pregnancy could increase the risk of term low birth weight (LBW), while the susceptible window of the pollutants varied [3]. The risk of pregnancy-induced hypertension (PIH) syndrome is not only related to the air pollutants and concentrations but also closely related to different trimesters [4]. Meanwhile, the risk of premature rupture of membranes (PROM) could be increased by underlying infection, inflammation, oxidative stress, nutritional deficiencies, cigarette smoking, air pollutants’ exposure, and illicit drug use [5,6,7,8].

Apart from the adverse effects on pregnant women [4], smog pollution directly affects infants and has a long-term effect on their health conditions when they grow up, including hypertension [9], cardiac disease [10], and type 2 diabetes mellitus [11]. However, studies investigating the association of smog pollution with birth outcomes only considered either pregnant mothers or their offspring [12,13,14,15], few of them focused on both sides [16], and the results were inconsistent and controversial [3]. Furthermore, relative studies involving Chinese population are limited and lagged.

With this background, we performed a population-based retrospective study in Baoding, Hebei, a region with serious fog and haze pollution in China [17], to examine the effects of smog pollutants on the risk of birth outcomes of both pregnant mothers and their offspring to identify susceptible exposure windows. Given the cross-region and cross-basin smog pollution [18], this study provides valuable evidence for other pollution-exposed areas.

## 2 Materials and methods

### 2.1 Smog pollutants

From October 2013 to October 2016, the ongoing population-based retrospective study was conducted mainly to investigate the impact of environmental factors on pregnant outcomes. Data on smog pollutants were obtained from the Baoding Environmental Protection Bureau, located in Baoding, Hebei, China. This bureau is a subordinate unit of the Ministry of Ecology and Environment of the People’s Republic of China, which is responsible for the supervision and administration of environmental pollution prevention and control. An automated data reporting system equipped with satellite remote sensing, meteorologic, and land use information was used to collect the 24 h average concentration of six kinds of smog pollutants, namely, PM2.5, inhalable particle (particulate matter with aerodynamics diameter <10 μm) (PM10), sulfur dioxide (SO_2_), nitrogen dioxide (NO_2_), carbon monoxide (CO), and ozone (O_3_). Median was used to represent the average concentration of individual pollutants during different trimesters, and the category of air quality index (AQI) that corresponded to the median value was used for the statistical analysis. AQI was categorized into good, mild pollution, moderate pollution, and above (Tables A1 and A2). Classification of pollutants in the current study is based on the degree of its impact on human health. “Good” means that the air had minimal effect on healthy population, “mild pollution” indicates that pollution caused irritation symptoms in healthy population, and “moderate pollution” means that it affects the heart or respiratory system in healthy population.

### 2.2 Study population

We limited the study population to the resident population in Baoding, which is close to the pollutant monitoring station. This study obtained participants’ residence information from the registration of medical records specific to the street and doorplate numbers. The duration of data collection was the same with the data of smog pollutants.

**Figure 1:**
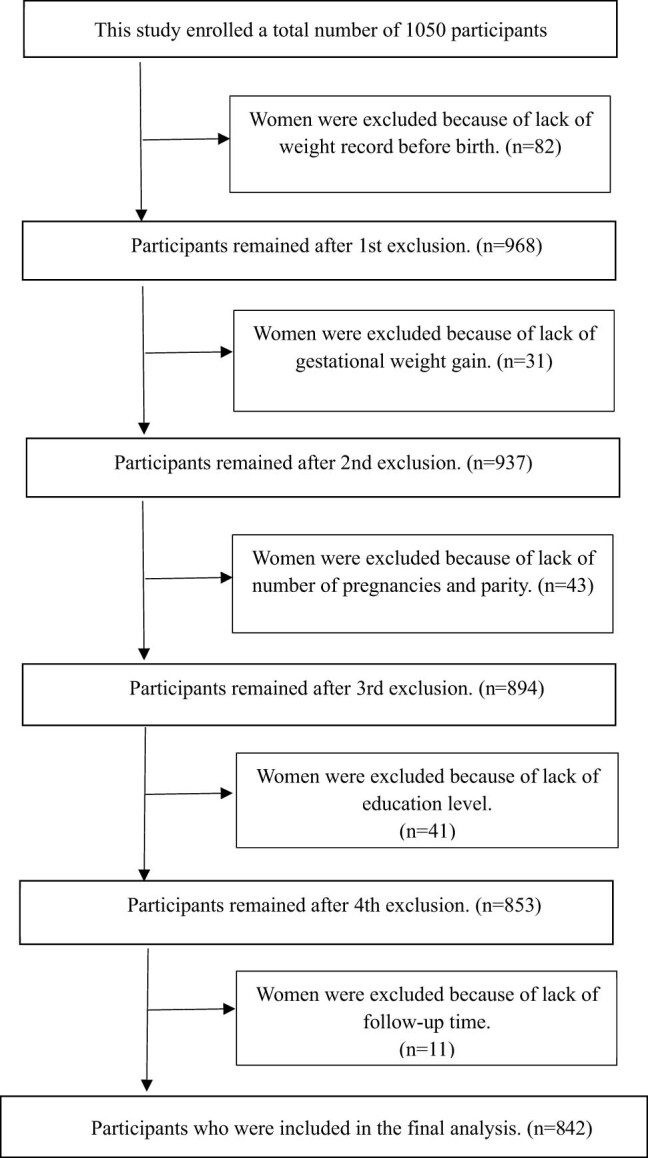
Process about inclusion and exclusion of participants.

The clinical data were obtained from the electronic medical records system. A total of 1,050 participants were enrolled in this study. Among the 1,050 patients, 208 were excluded due to the lack of weight record before birth (*n* = 82), gestational weight gain (*n* = 31), number of pregnancies and parity (*n* = 43), education level (*n* = 41), and follow-up time (*n* = 11). Finally, 842 women were included in the statistical analysis (Figure 1). Given that the number of individuals with comorbidity was relatively small (<2% of the sample size), individuals with comorbidity were excluded in the final statistical analysis. Participants included were all term singleton live birth born (37 ≤ gestational weeks < 42). The participant’s number (prevalence) of term LBW, PIH, GDM, and PROM was 69 (8.2%), 125 (14.8%), 139 (16.5%), and 102 (12.1%), respectively. The first trimester of pregnancy was defined as gestational week 1 to week 12, the second trimester was defined as week 12^+1^ to week 27, and the third trimester of pregnancy was defined as from week 27^+1^ to birth [16].

Maternal age (20–24, 25–29, 30–34, and ≥35) [19], gestational weight gain (appropriate weight gain, insufficient weight gain, or excessive weight gain), pre-pregnancy body mass index (BMI) (low body weight, normal type, overweight, or obesity), education level (<high school, high school/polytechnic school, college, or above), last menstrual date, delivery date, number of pregnancies (1, 2, or ≥3 times), and parity (1, 2, or ≥3 times) were included in the study. According to the American Academy of Medical Science [Institute of Medicine (IOM)] [20], the range of gestational weight gain for low-body-weight (BMI < 18.5 kg/m^2^) women is 12.5–18.0 kg, the weight gain for normal-type (18.5 kg/m^2^ ≤ BMI ≤ 24.9 kg/m^2^) women is 11.5–16.0 kg, the weight gain for overweight (25 kg/m^2^ ≤ BMI ≤ 29.9 kg/m^2^) women is 7.0–11.5 kg, and the weight gain for women with obesity (BMI ≥ 30 kg/m^2^) is 5.0–9.0 kg. In different BMI groups, gestational weight gain was appropriate when it was within the recommended range. People who had weight values below the recommended range had insufficient weight gain. By contrast, people who had weight values above the recommended range had excessive weight gain [20].

### 2.3 Observed outcomes

The outcomes of LBW, PIH, GDM, and PROM were defined on the basis of disease classification by the International Classification of Diseases, Tenth Revision. Term LBW is defined as a birth that occurred on or after the 37th week of gestation with weight <2,500 g [21]. PIH is defined as blood pressure ≥140/90 mm Hg manifested initially during pregnancy and normalized at 12 weeks postpartum [22]. PIH included pregnancy hypertension, preeclampsia, and eclampsia in this study. Preeclampsia is defined as gestational hypertension accompanied by proteinuria after 20 weeks of gestation, characterized by proteinuria and hypertension [23]. Eclampsia is defined as convulsions occurring on the basis of preeclampsia that cannot be explained by other causes. GDM refers to the first clinical manifestation of gestational diabetes caused by abnormal glucose metabolism after pregnancy [24]. Rupture of membranes before labor is defined as term PROM. PROM at gestational age <37 weeks is defined as premature birth or preterm PPROM, whereas PROM >37 weeks of gestation is defined as term PROM [22]. This current study aimed at analyzing term PROM.


**Ethics approval:** The current study was reviewed and approved by the Ethics Committee of the First Hospital of Hebei Medical University (Approval number: 20180701).

### 2.4 Statistical analysis

All analyses were performed using the SPSS software version 21.0 (SPSS Inc., Chicago, IL, USA). Categorical variables were described as frequency (percentage) and were analyzed with chi-square tests. An unconditional binary logistic regression model was used to calculate the odds ratios (ORs) and 95% confidence intervals (CIs) for associations between smog pollutant exposure during pregnancy period and risk of adverse birth outcomes adjusting for maternal age, gestational weight gain, pre-pregnancy BMI, education level, and number of pregnancies and parity. We examined the association by the following different exposure windows: entire pregnancy, trimester 1, trimester 2, and trimester 3. All statistical tests were two-sided, and *P* values <0.05 were statistically significant.

## 3 Results

### 3.1 Characteristics at baseline of birth outcomes

The characteristics at baseline of participants are summarized in Table 1. In total sample, nearly half of the pregnant women were from 25 to 29 years of age, and women over 35 accounted for the smallest percentage of the participants. The proportions of appropriate weight gain and excessive weight gain during pregnancy accounted for the largest. Nearly 10 percent of the participants were under low body weight before pregnancy, while the pre-pregnancy BMIs of most individuals were within the normal range. The differences in education level were obvious, which showed that the low-education level (<high school) and the high-education level (college or above) coexist. The distribution of number of pregnancies was relatively even, with about a third of pregnancies in each category. The proportions of parity in 1 and 2 accounted for over 90%. Most of the participants with adverse birth outcomes were under 30 years old and experienced excessive weight gain during pregnancy. Many individuals were overweight or obese before pregnancy, except for cases in PROM. The participants who underwent GDM and PROM had relatively higher-education levels. When the parity increased, the incidence of LBW, PIH, and PROM decreased.

**Table 1:** The characteristics at baseline of birth outcomes *n* (%)

Characteristics	Total sample	LBW	PIH	GDM	PROM
Age (years)					
20–24	148 (17.6%)	15 (9.4)	28 (17.6)	20 (12.6)	25 (15.7)
25–29	380 (45.1%)	30 (7.9)	42 (11.1)	54 (14.2)	55 (14.5)
30–34	199 (23.6%)	15 (7.5)	32 (16.1)	32 (16.1)	9 (4.5)
≥35	104 (12.4%)	9 (8.7)	23 (22.1)	33 (31.7)	3 (12.5)
Gestational weight gain					
Appropriate weight gain	337 (40.0%)	22 (7.3)	45 (14.9)	45 (14.9)	43 (14.2)
Insufficient weight gain	140 (16.6%)	11 (8.8)	11 (8.8)	20 (16.0)	15 (12.0)
Excessive weight gain	365 (43.3%)	36 (8.7)	69 (16.7)	74 (17.9)	44 (10.6)
Pre-pregnancy BMI					
Normal type	544 (64.6%)	32 (6.9)	49 (10.5)	64 (13.7)	62 (13.3)
Low body weight	86 (10.2%)	8 (9.3)	7 (8.1)	8 (9.3)	15 (17.4)
Overweight or obesity	212 (25.2%)	29 (10.0)	69 (23.9)	67 (23.2)	25 (8.7)
Education Level					
<High school	376 (44.7%)	44 (13.3)	77 (23.3)	46 (13.9)	39 (11.8)
High school/polytechnic school	77 (9.1%)	8 (6.5)	15 (12.2)	15 (12.2)	15 (12.2)
College or above	389 (46.2%)	17 (4.4)	33 (8.5)	78 (20.1)	48 (12.3)
Number of pregnancies					
1	320 (38.0%)	26 (8.1)	40 (12.5)	45 (14.1)	59 (18.4)
2	237 (28.1%)	19 (8.1)	40 (16.9)	41 (17.4)	20 (8.5)
≥3	285 (33.8%)	24 (8.4)	45 (15.8)	53 (18.6)	23 (8.1)
Parity					
≤1	446 (53.0%)	38 (8.5)	62 (13.9)	63 (14.1)	77 (17.3)
2	322 (38.2%)	25 (7.8)	52 (16.1)	64 (19.9)	21 (6.5)
≥3	74 (8.8%)	6 (8.1)	11 (14.9)	12 (16.2)	4 (5.4)

Abbreviations: LBW: low birth weight, PIH: pregnancy-induced hypertension syndrome, GDM: gestational diabetes mellitus, PROM: premature rupture of membranes.

### 3.2 Correlations between covariables and outcomes

Among the covariables, only the education level was related to LBW. The risk of term LBW gradually decreased with the increase in the education level in the entire pregnancy and the three trimesters (Table 2). The risk of PIH gradually decreased with the education level and increased with the pre-pregnancy BMI in the entire pregnancy and the three trimesters (Tables 3 and 4). Meanwhile, the risk of term PROM gradually decreased with the parity number during trimester 3 (Table 5).

**Table 2:** The correlations between education levels and term low birth weight (OR, 95% CI)

	< High school	High school/polytechnic school	College or above	*P*-value
Entire pregnancy	1.00	0.366 (0.157, 0.857)	0.300 (0.166, 0.544)	<0.0001
Trimester 1	1.00	0.358 (0.153, 0.837)	0.305 (0.169, 0.551)	<0.0001
Trimester 2	1.00	0.451 (0.206, 0.987)	0.296 (0.166, 0.529)	<0.0001
Trimester 3	1.00	0.463 (0.211, 1.017)	0.311 (0.173, 0.559)	<0.0001

**Table 3:** The correlations between education levels and pregnancy-induced hypertension syndrome (OR, 95% CI)

	< High school	High school/polytechnic school	College or above	*P*-value
Entire pregnancy	1.00	0.454 (0.241, 0.856)	0.336 (0.214, 0.527)	<0.0001
Trimester 1	1.00	0.434 (0.229, 0.823)	0.335 (0.214, 0.526)	<0.0001
Trimester 2	1.00	0.507 (0.275, 0.935)	0.338 (0.215, 0.530)	<0.0001
Trimester 3	1.00	0.526 (0.285, 0.971)	0.357 (0.228, 0.561)	<0.0001

**Table 4:** The correlations between pre-pregnancy BMI and pregnancy-induced hypertension syndrome (OR, 95% CI)

	Normal weight	Low body weight	Overweight or obesity	*P*-value
Entire pregnancy	1.00	0.737 (0.314, 1.728)	2.273 (1.498, 3.451)	<0.0001
Trimester 1	1.00	0.799 (0.343, 1.857)	2.496 (1.641, 3.787)	<0.0001
Trimester 2	1.00	0.740 (0.318, 1.718)	2.458 (1.626, 3.717)	<0.0001
Trimester 3	1.00	0.687 (0.294, 1.603)	2.260 (1.491, 3.425)	<0.0001

**Table 5:** The correlations between parity and term premature rupture of membranes in trimester 3 (OR, 95% CI)

Parity number	≤1	2	≥3	*P*-value
Trimester 3	1.00	0.348 (0.198, 0.610)	0.294 (0097, 0.885)	<0.0001

### 3.3 Smog pollutants and maternal and fetal birth outcomes

The distribution of cases exposed to the pollutants at different trimesters is summarized in Table 6. The composition of pollutants varied among different trimesters, and the most serious pollutants were PM2.5 and PM10. Compared with “good” condition, exposure to mild pollution of PM2.5 and PM10 significantly increased the risk of term LBW during the entire pregnancy. The risk of LBW gradually increased as the pollution of PM2.5 worsened during trimester 1. Meanwhile, CO in mild pollution significantly increased such risk during trimester 3 (Table 7).

**Table 6:** Distribution of case exposed to various pollutants [*n* (%)]

Pollutants	Category of AQI	LBW	PIH	GDM	PROM	Pollutants	Category of AQI	LBW	PIH	GDM	PROM
**Entire pregnancy**						**Trimester 2**					
PM2.5	Good	21 (4.6)	47 (10.2)	77 (16.7)	51 (11.1)	PM2.5	Good	30 (9.6)	59 (18.8)	45 (14.3)	54 (17.2)
	Mild pollution	48 (12.6)	78 (20.5)	62 (16.3)	51 (13.4)		Mild pollution	16 (10.1)	23 (14.6)	26 (16.5)	13 (8.2)
PM10	Good	62 (7.5)	118 (14.2)	136 (16.4)	102 (12.3)		Moderate pollution and above	23 (6.2)	43 (11.6)	68 (18.4)	35 (9.5)
	Mild pollution	7 (53.8)	7 (53.8)	3 (23.1)	0 (0)	PM10	Good	32 (9.6)	62 (18.5)	47 (14.0)	55 (16.4)
SO_2_	Good	69 (8.2)	125 (14.8)	139 (16.5)	102 (12.1)		Mild pollution	37 (7.3)	43 (11.6)	92 (18.1)	47 (9.3)
NO_2_	Good	69 (8.2)	125 (14.8)	139 (16.5)	102 (12.1)	SO_2_	Good	69 (8.2)	125 (14.8)	139 (16.5)	102 (12.1)
CO	Good	69 (8.2)	125 (14.8)	139 (16.5)	102 (12.1)	NO_2_	Good	64 (8.2)	112 (14.3)	130 (16.6)	98 (12.5)
O_3_	Good	69 (8.2)	125 (14.8)	139 (16.5)	102 (12.1)		Mild pollution	5 (8.2)	13 (21.3)	9 (14.8)	4 (6.6)
**Trimester 1**						CO	Good	69 (8.2)	125 (14.8)	139 (16.5)	102 (12.1)
PM2.5	Good	54 (7.1)	109 (14.2)	127 (16.6)	94 (12.3)	O_3_	Good	69 (8.2)	125 (14.8)	139 (16.5)	102 (12.1)
	Mild pollution	7 (11.1)	7 (11.1)	10 (15.9)	6 (9.5)	**Trimester 3**					
	Moderate pollution and above	8 (57.1)	9 (64.3)	2 (14.3)	2 (14.3	PM2.5	Good	35 (7.1)	58 (11.7)	90 (18.2)	44 (8.9)
PM10	Good	54 (7.0)	110 (14.2)	129 (16.6)	95 (12.3)		Mild pollution	7 (21.2)	13 (39.4)	6 (18.2)	5 (15.2)
	Mild pollution	15 (22.4)	15 (22.4)	10 (14.9)	7 (10.4)		Moderate pollution and above	27 (8.6)	54 (17.2)	43 (13.7)	53 (16.9)
SO_2_	Good	69 (8.2)	125 (14.8)	139 (16.5)	102 (12.1)	PM10	Good	39 (7.7)	62 (12.2)	91 (17.9)	48 (9.4)
NO_2_	Good	66 (7.9)	121(14.5)	138(16.5)	102(12.2)		Mild pollution	28 (8.5)	62 (18.8)	47 (14.3)	51 (15.5)
	Mild pollution	3 (50.0)	4 (66.7)	1 (16.7)	0 (0)		Moderate pollution and above	2 (40.0)	1 (20.0)	1 (20.0)	3 (60.0)
CO	Good	69 (8.2)	125 (14.8)	139 (16.5)	102 (12.1)	SO_2_	Good	69 (8.2)	125 (14.8)	139 (16.5)	102 (12.1)
O_3_	Good	69 (8.2)	125 (14.8)	139 (16.5)	102 (12.1)	NO_2_	Good	51 (7.6)	87 (13.0)	110 (16.5)	75 (11.2)
							Mild pollution	18 (10.3)	38 (21.7)	29 (16.6)	27 (15.4)
						CO	Good	66 (7.9)	121 (14.5)	137 (16.4)	99 (11.9)
							Mild pollution	3 (37.5)	4 (50.0)	2 (25.0)	3 (37.5)
						O_3_	Good	69 (8.2)	125 (14.8)	139 (16.5)	102 (12.1)

Abbreviations: LBW: low birth weight, PIH: pregnancy-induced hypertension syndrome, GDM: gestational diabetes mellitus, PROM: premature rupture of membranes.

PM2.5: fine particulate matter (particulate matter with aerodynamics diameter less than 2.5 μm), PM10: inhalable particle (particulate matter with aerodynamics diameter less than 10 μm), SO_2_: sulfur dioxide, NO_2_: nitrogen dioxide, CO: carbon monoxide, O_3_: ozone.

**Table 7:** The effect of smog pollutants on LBW, PIH, and PROM (OR and 95% CI)

	Good	Mild pollution	Moderate pollution and above	*P*-value
**LBW**				
Entire pregnancy				
PM2.5	1.00	2.60 (1.50–4.51)	—	0.001
PM10	1.00	10.50 (3.15–35.01)	—	<0.001
Trimester 1				
PM2.5	1.00	1.55 (0.67–3.62)	18.97 (5.97–60.32)	<0.001
Trimester 3				
CO	1.00	4.55 (1.02–19.40)	—	0.047
**PIH**				
Entire pregnancy				
PM2.5	1.00	1.96 (1.30–2.95)	—	0.001
PM10	1.00	5.15 (1.58–16.77)	—	0.007
Trimester 1				
PM2.5	1.00	0.74 (0.32–1.70)	12.09 (3.73–39.17)	<0.001
Trimester 2				
PM10	1.00	0.58 (0.38–0.89)	—	0.012
NO_2_	1.00	2.39 (1.17–4.85)	—	0.016
Trimester 3				
PM2.5	1.00	3.40 (1.53–7.53)	1.44 (0.95–2.18)	0.006
**PROM**				
Trimester 3				
PM10	1.00	1.72 (1.11–2.65)	18.82 (2.69–131.45)	0.001

Abbreviations: LBW: low birth weight, PIH: pregnancy-induced hypertension syndrome, GDM: gestational diabetes mellitus, PROM: premature rupture of membranes.

PM2.5: fine particulate matter (particulate matter with aerodynamics diameter less than 2.5 μm), PM10: inhalable particle (particulate matter with aerodynamics diameter less than 10 μm), SO_2_: sulfur dioxide, NO_2_: nitrogen dioxide, CO: carbon monoxide, O_3_: ozone.

When pregnant women were exposed to mild pollution of PM2.5 and PM10 during the entire pregnancy, PIH risk significantly increased compared with those in “good” condition. The risk also significantly increased by mild pollution of PM10 and NO_2_ during trimester 2. Mild pollution, moderate pollution, and above of PM2.5 also increased the risk of PIH during trimester 1 and trimester 3 (Table 7).

The risk of term PROM gradually increased when PM10 pollution worsened during trimester 3. Pregnant women were more at risk of experiencing term PROM by 1.72 times when exposed to moderate pollution and by 18.82 times when exposed to moderate pollution and above than those participants in “good” condition (Table 7).

No correlation between smog pollutants and GDM was found (Table 8).

**Table 8:** Effect of factors on GDM (four trimesters)^1^

Factors	OR (95% CI)	*P*-value
Age		
20–24	1.00	0.006
25–29	0.94 (0.53, 1.67)	
30–34	0.98 (0.52, 1.84)	
≥35	2.27 (1.18, 4.36)	
Pre-pregnancy BMI		
Normal type	1.00	0.004
Low body weight	0.67 (0.31, 1.46)	
Overweight or obesity	1.79 (1.20, 2.67)	
Education level		
<High school	1.00	0.019
High school/polytechnic school	0.88 (0.46, 1.66)	
College or above	1.67 (1.11, 2.57)	

Abbreviation: GDM: gestational diabetes mellitus.

^1^Results in trimester 1, trimester 2, and trimester 3 were consistent with those during entire pregnancy.

## 4 Discussion

**Figure 2:**
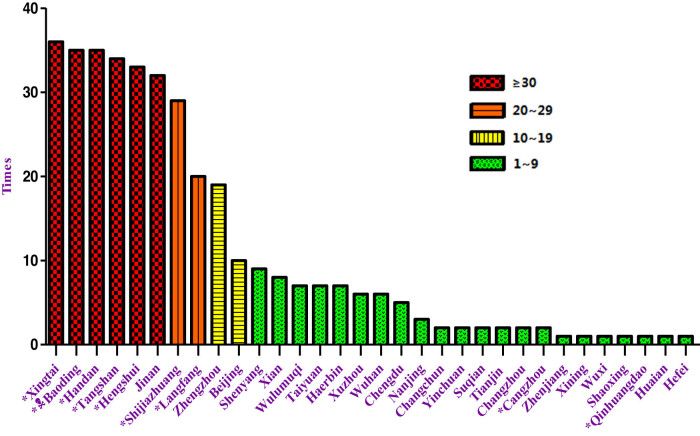
Frequency chart of the 10 most seriously polluted cities according to the ranking of Ambient Air Comprehensive Pollution Index (2013.10–2016.10). According to the ranking of Ambient Air Comprehensive Pollution Index of 74 major cities in China from October 2013 to October 2016, issued by the Ministry of Ecological Environment of the People’s Republic of China, 10 most seriously polluted cities were counted for 3 years (36 months). Totally, 32 cities have been ranked in most seriously polluted cities during 36 months. In these 32 cities, nine cities are affiliated with Hebei, accounting for nearly 30%. Baoding entered 35 times in the chart of most seriously polluted cities, ranking No. 2. *The city belonging to Hebei.

We employed an estimation of six components of smog pollutants (PM2.5, PM10, SO_2_, NO_2_, CO, and O_3_) to examine the associations between four outcomes (term LBW, PIH, GDM, and PROM) in Baoding, Hebei, China, from 2013 to 2016. PM concentrations in many developing countries (e.g., India and China) are 5–10 times higher than in developed countries [25]. Hebei is a province with serious fog and haze pollution in China [26]. According to the ranking of Smog Comprehensive Pollution Index of 74 major cities in China, from October 2013 to October 2016, 32 cities were ranked as the most seriously polluted cities during 36 months [27]. In these 32 cities, nine are affiliated with Hebei, and Baoding ranks second (Figure 2).

Fleischer et al. investigated the association of satellite-based estimates of PM2.5 and preterm birth and LBW (all gestational ages) by using the WHO Global Survey on Maternal and Perinatal Health in Africa, Asia, and Latin America [1]. In China, LBW was associated with the 3rd and 4th quartiles of PM2.5 (OR = 1.08; 95% CI: 0.84, 1.40; and OR = 1.99; 95% CI: 1.06, 3.72) [1]. An increase in the concentration of PM2.5 reduced the term birth weight during the entire pregnancy [28], thereby conforming to our results. In the present study, the risk of term LBW gradually increased with the increase of PM2.5 concentrations during the entire pregnancy and trimester 1. In addition, with the increase of PM10 concentrations, the risk of LBW under mild pollution was 10.5 times higher than that in good condition during the entire pregnancy. Other researchers also found that PM10 at 10 μg/m^3^ increments in trimester 2 led to decreases in birth weight of 5.65 g [29]. Meanwhile, the risk of term LBW increases by 4.55 times with the increase in CO concentrations during trimester 3, as supported by the study of Li et al. [3]. In general, the risk of LBW in Baoding was higher than that in China, which suggests that more effective environmental protection measures should be taken to protect pregnant women, especially in the North area where severe air pollution exists.

PM2.5 and preeclampsia, which is one disease of PIH, are positively associated [4,30]. Similar with Mobasher’s results [4], the current study found that exposures to PM2.5 at trimester 1 significantly increase the PIH. In addition to trimester 1, this disadvantageous effect was observed during the entire pregnancy and trimester 3. Low concentrations of PM2.5 and PM10 did not increase the risk of PIH in trimesters 1 and 2 due to the low incidence of PIH within these trimesters. However, the harmful effects to health were aggravated, and the risk of PIH escalated when PM2.5 concentration increased. Besides, the current study also found that pregnant women in trimester 3 are more sensitive to PM2.5 pollution, and the risk of PIH increased in this period. A study performed by Bai et al. found that PM10 exposure is associated with an increased risk of PIH [31]. In the present study, the risk of PIH increased with the increase of PM10 concentration during the entire pregnancy, not during trimester 2. In addition, pregnant women were more susceptible to NO_2_ exposure during trimester 2, resulting in an increased risk of PIH in this term. Thus, the risk of PIH was not only related to the air pollutants and the concentrations but also closely related to different trimesters.

In the present study, the risk of term PROM gradually increased with the increase in PM10. Wallace et al. reported that PM10 and PROM have a negative correlation [32]. The discrepancy might be explained by the concern on term PROM as a birth outcome in the current study, whereas the outcomes involved in their study were PROM at any gestational period and PPROM. We focused on term PROM for the following reasons. Approximately 70% of PROM occur at term, which is the cause of approximately one-third of all preterm births [33]. Term PROM is a significant cause of perinatal morbidity and mortality [33]. We also studied the relationship between PM10 and PROM during other periods of pregnancy, and no significant relationship existed between them (data not shown). Despite these suggested associations, the specific mechanism between air pollution and PROM remains unclear, and further studies were needed to shed light on potential mechanisms.

Silvestrin et al. found that high maternal education showed a 33% protective effect against LBW [34]. The current results were similar with this finding in which the risk of term LBW gradually decreased with the increase in the education level in all trimesters. Maternal education is a suitable variable to measure inequality in health care and has been used to assess birth outcomes [35,36]. As extensively studied worldwide, education is the strongest socioeconomic predictor of health status and is the most important determinant of birth weight in a population [37].

Seung Chik Jwa found that the low-education-level group had higher systolic and diastolic blood pressure levels in the early pregnancy. However, the same associations were not found after adjusting for pre-pregnancy BMI [38]. The current study found that education level indicated a protective effect on the risk of PIH during the entire pregnancy and during trimesters 1, 2, or 3. The risk of PIH decreased with the increase in the education level. Moreover, the conclusion was based on the correction of all the confounding factors, which include the pre-pregnancy BMI. People with high-education levels are concentrated on healthy lifestyle, eating habits, and prenatal checkups, which should be reasonable and standardized. This statement might be the reason for the current findings above. According to Amoakoh-Coleman et al., pregnant women who were obese at baseline had a threefold increased risk of PIH compared with which with normal BMI [Relative risk (RR) = 3.01 (1.06–8.52), *P* = 0.04] [39]. The current study confirmed this result and showed evidence that the risk of PIH gradually increased with the increase in pre-pregnancy BMI during the entire pregnancy and during trimesters 1, 2, or 3.

The current study also revealed that parity is a protective factor for term PROM, resulting in the gradual decrease in the risk of term PROM as parity increased (OR = 0.294; 95% CI: 0.097, 0.885), conforming to the study accomplished by Jiang et al. in Beijing [40].

No association between smog pollutants and GDM was found in this current study. However, the risk of GDM gradually increased with the increase in pre-pregnancy BMI during the entire pregnancy and individual three trimesters. Dave found that BMI ≥25 kg/m^2^ is a strong risk factor for GDM [41]. In the present study, age also increased the risk of GDM. The risk of GDM in >35-year-old women was 2.27 times higher than that in 20–24-year-old women. A survey from Korea also implies that older maternal age is associated with the development of GDM [42]. The fact that women with higher-education level had a higher risk of GDM was linked to be their later pregnancy and older age. More research should be carried out to clarify the role of pollution in the risk of GDM.

It is more comprehensive to focus on the adverse pregnancy outcomes of both pregnant mothers and the newborns in the present study. And the city we concerned could be regarded as a representative of cities with serious air pollution in North China. The results may shed light on pregnant women’s health, medical institutions’ rational resource allocations, and decision-makers’ choices of environmental measures.

There are several limitations in this study. First, the lack of available information regarding physical activity, nutritional status, smoking, and alcohol consumption might have effects on the association between smog and birth outcomes. Second, this study was an observational, single-centered study. Further studies with multi-city, multi-center, and larger samples are needed for more evidence.

## 5 Conclusion

In this population-based retrospective study, the susceptible exposure windows between smog pollutants and the risk of birth outcomes were revealed. Compared with the lower pollution level, the higher pollution level of PM2.5, PM10, and CO increased the risk of term LBW during trimester 1, trimester 3, and the entire pregnancy. PM2.5, PM10, and NO_2_ increased the risk of PIH during different trimesters, while PM10 increased the risk of PROM during trimester 3. The findings of our analysis may help decision-makers to develop targeted policies and environmental measures to reduce the health hazards of air pollution.

## Appendix

**Table A1:** Individual air quality index and corresponding pollutants concentration limits (24h mean concentration)*

AQI	SO_2_ (μg/m^3^)	NO_2_ (μg/m^3^)	PM10 (μg/m^3^)	CO (mg/m^3^)	O_3_ (μg/m^3^)	PM2.5 (μg/m^3^)
0	0	0	0	0	0	0
50	50	40	50	2	160	35
100	150	80	150	4	200	75
150	475	180	250	14	300	115
200	800	280	350	24	400	150

***** Extracted from the Environmental Air Quality Index (AQI) Technical Regulations (for Trial Implementation) (HJ633 to 2012) issued by the Ministry of Environmental Protection of the People's Republic of China.

Abbreviations: PM2.5: fine particulate matter (particulate matter with aerodynamics diameter less than 2.5 μm), PM10: inhalable particle (particulate matter with aerodynamics diameter less than 10μm), SO_2_: sulfur dioxide, NO_2_: nitrogen dioxide, CO: carbon monoxide, O_3_: ozone.

**Table A2:** Air quality index and impact on health*

Level (AQI)	Category ^#^	Impact on health
≤2nd degree (≤100)	Good	Some pollutants have delicate effect on healthy population except for a very few extremely sensitive people.
3rd degree (101–150)	Mild Pollution	Irritation symptoms in healthy population.
4th degree (151–200)	Moderate Pollution	May be have an impact on the heart or respiratory system in healthy population.

*Extracted from the Environmental Air Quality Index (AQI) Technical Regulations (for Trial Implementation) (HJ633 to 2012) issued by the Ministry of Environmental Protection of the People's Republic of China.

*Concentrations of various smog pollutants were divided into different air quality index categories according to their corresponding air quality index (AQI). The category of air quality index was included in statistical analysis in current study.
